# Biology, Therapy and Implications of Tumor Exosomes in the Progression of Melanoma

**DOI:** 10.3390/cancers8120110

**Published:** 2016-12-09

**Authors:** Allison L. Isola, Kevinn Eddy, Suzie Chen

**Affiliations:** 1 Susan Lehman Cullman Laboratory for Cancer Research, Ernest Mario School of Pharmacy, Rutgers, the State University, Piscataway, NJ 08854, USA; allisonisola@gmail.com (A.L.I.); kevinneddy@gmail.com (K.E.); 2 Joint Graduate Program in Toxicology, Rutgers, The State University, Piscataway, NJ 08854, USA; 3 Rutgers Cancer Institute of New Jersey, New Brunswick, NJ 08901, USA

**Keywords:** cancer, melanoma, metastasis, exosomes, melanoma exosomes

## Abstract

Cancer is the second leading cause of death in the United States, and about 6% of the estimated cancer diagnoses this year will be melanoma cases. Melanomas are derived from transformation of the pigment producing cells of the skin, melanocytes. Early stage melanoma is usually curable by surgical resection, but late stage or subsequent secondary metastatic tumors are treated with some success with chemotherapies, radiation and/or immunotherapies. Most cancer patients die from metastatic disease, which is especially the case in melanoma. A better understanding of tumor metastasis will provide insights and guide rational therapeutic designs. Recently, the importance of melanoma-derived exosomes in the progression of that cancer has become more apparent, namely, their role in various stages of metastasis, including the induction of migration, invasion, primary niche manipulation, immune modulation and pre-metastatic niche formation. This review focuses on the critical roles that melanoma exosomes play in the progression of this deadly disease.

## 1. Melanoma

Hanahan and Weinberg were the first to address the six biological hallmarks of cancer and they recently added four additional new hallmarks as necessary traits during the development and progression of cancer [1]. These hallmarks are: unregulated cell growth, anti-apoptosis signals, induction of angiogenesis, unresponsive to growth suppressors, metastatic capabilities, replicative immortality, genomic instability, immune system evasion, tumor-specific inflammatory response and transformation of cellular metabolism. Here we will use melanoma as an example to address one of the hallmarks: metastasis. Metastasis is the process by which cancer cells travel to distant locations throughout the body, via the circulatory system, and form secondary tumors, with the ability to survive on their own in a preferred site, which is cancer type dependent [2].

In the United States, it is estimated by the American Cancer Society that approximately 76,000 new cases of invasive melanoma will be diagnosed, and about 10,000 deaths will be attributed to melanoma in 2016 [3]. Melanoma patients only account for about 5% amongst all of the skin cancer cases, but it is responsible for the majority of deaths of skin cancer patients. If detected early, primary melanoma tumors are surgically removed, resulting in a five-year survival rate of 92% and a ten-year survival rate of 89% [3]. In late stage melanoma cases that have metastasized, the one-year survival rate drops to 35%–62% [4], with the most common sites of metastasis being the lungs and the brain [5].

## 2. Melanocytes

Melanoma is derived from melanocytes, the pigment-forming cells of the skin, hair follicles, uvea, inner ear, nervous system and heart [6]. Melanocytes originated from neural crest cells and have the ability to produce melanin through a specialized membrane bound organelle known as a melanosome [6,7]. Phenotypically, melanocytes are oval shaped, have dendritic arms and are typically 7 µm in diameter [6]. Dendritic arms found on melanocytes allow for cell-cell interaction with keratinocytes, enabling the transfer of melanin-containing melanosomes from the melanocytes to the keratinocytes [6,7]. This transfer of melanin to keratinocytes determines the color of the skin and hair of an individual. Melanin functions as perinuclear protection against harmful ultraviolet (UV) radiation in keratinocytes, store ions, as a free radical scavenger, and couples oxidation-reduction reactions [6,7]. Interestingly, melanin has been demonstrated to have detrimental effects in vitro in normal human melanocytes, where it was shown to enhance single stranded DNA breaks, which could be explained by the formation of reactive oxygen species (ROS) during photo-oxidation of melanin in experimental settings [7]. Results from these studies suggest that the complex functions of melanin may be cell type dependent.

## 3. Melanoma Types

Melanoma can be divided into two categories: non-cutaneous and cutaneous melanoma. Cutaneous melanoma is the most common type of melanoma, accounts for up to 91.2% of all melanoma cases and is thought to be caused by genetic predisposition or unrepaired DNA damage due to environmental factors including UV radiation exposure. Non-cutaneous melanoma arises from transformed melanocytes located near the eyes (5.2%) and the mucosal tissues (1.3%) such as the genital, anal, esophageal, nasal and oral cavities [8,9]. Non-cutaneous melanomas are rare (less than 10% of all melanomas) when compared to cutaneous melanomas [8].

There are four sub-types of cutaneous melanoma: acral melanoma, mucosal melanoma, chronic sun-induced damaged (CSID) and non-chronic sun-induced damage (NCSID) [10]. Acral and mucosal melanomas have significantly more chromosomal aberrations such as increases or decreases in copy number of specific chromosomal regions when compared to cutaneous melanoma [9,10]. Acral melanoma is commonly found in individuals with darker skin such as Africans, Asians and Hispanics and is commonly associated with melanocytes of the palms, soles and mucosal surfaces [8,9].

There have been numerous molecular pathways that are commonly dysregulated in melanoma. The most common ones are the MAPK and PI3K/AKT pathways. In the MAPK pathway, common mutated proteins include RAS and RAF. This pathway is highly conserved and when activated by upstream signaling cascades [such as growth factor receptors and G-protein coupled receptors (GPCRs)], leads to cell proliferation, differentiation and migration. Aberrant activation of this pathway by some of the mutations in components within the pathway is frequently seen in melanoma. An example of this is a single-base missense transversion causes the replacement of valine with glutamic acid at amino acid residue 600 in BRAF that is detected in about 85% of nevi and melanoma [11,12]. BRAF is a serine/threonine kinase that is part of the MAPK signaling cascade found downstream of RAS; it activates MEK by phosphorylation, which in turn activates ERK also by phosphorylation. Mutated BRAF not only upregulates its own kinase activity, but also that of MEK and ERK, and promote cell proliferation [13]. In addition to mutated BRAF, mutated NRAS accounts for about 20% of melanoma cases [14].

In uveal melanoma it has been shown that a mutation in the GTP binding region of the Gα subunit (Q209L) blocks the cleavage of GTP to GDP and deregulates both MAPK and PI3K/AKT pathways [15]. Transgenic mice harboring this mutated Gα subunit have increased skin lesions [15].

Our laboratory has been studying one of the upstream components of MAPK signaling cascade, a GPCR. We demonstrated that ectopic expression of metabotropic glutamate receptor 1 (GRM1) in melanocytes was sufficient to induce spontaneous melanoma development in vivo and transformation in vitro [16,17]. Interestingly, the ectopic expression of GRM1 mediated melanomagenesis is independent of the genotype of either BRAF or NRAS [18]. The precise mechanism by how the aberrant expression of GRM1 in melanocytes leads to oncogenesis remains unknown.

GRM can be subdivided into three groups, group I, II, and III based on sequence homologies and second messengers. Binding of the natural ligand, glutamate, to the receptor leads to phospholipase C (PLC) activation via G-protein subunits G_αq_/G_α11_ [19], allowing for the cleavage of phosphatidylinositol-4, 5-diphosphate (PIP2) to the production of two second messengers, inositol 1,4,5-triphosphate (IP3) and diacyl glycerol (DAG) [20]. IP3 then diffuses into the cytosol while DAG is bound to the membrane. The localization of IP3 within the cytosol results in the interaction with the endoplasmic reticulum leading to an increase in calcium concentration within the cytosol. Elevated calcium levels and interaction of the membrane bound DAG with protein kinase C (PKC), activates PKC’s kinase activity, which regulates cellular activity including MAPK [20]. Group II and III GRMs are coupled to the intracellular G-protein subunit, G_αi_/_o_, which mediates the inhibition of adenylyl cyclase [19,20], therefore, with activation of group II or III GRMs, there is a reduction in cAMP formation [19,20].

Calcium and DAG activate PKC, which, in turn, activates the MAPK and PI3K/AKT pathways allowing for the upregulation of cell proliferation and anti-apoptosis signals [21]. Additionally, activated PI3K/AKT pathway functions in tumor cell survival, epithelial mesenchymal transition (EMT), and angiogenesis. The MAPK pathway plays important roles in melanoma pathogenesis [19]. Melan-A cells, normal immortalized mouse melanocytes, showed increased cellular proliferation upon introduction of exogenous mutated BRAF (V600E), however no tumor formed [22]. In contrast, introduction of exogenous GRM1 into melan-A cells resulted in cell transformation in vitro and robust tumor formation in vivo [23]. Recently we also showed that in an in vivo mouse model with conditioned mutated BRAF (V600E), GRM1 expression is detected after mutated BRAF expression is activated, however, no tumor was detected in these mice up to 17 months of age. These preliminary results suggest that the temporal expression of mutated BRAF and GRM1 is critical in the determination of future tumorigenesis.

In addition to MAPK as one of the drivers for melanoma, there are other pathways that contribute to melanoma pathogenesis. The PI3K/AKT pathway also can contribute to pathogenesis, as a consequence of mutations or loss in PTEN and dysregulation in expression of AKT, which positively regulates the G1/S phase progression in cell cycle, suppresses apoptosis and promotes cellular survival. In a mutated BRAF mouse model, ablation of PTEN led to melanoma development [24], suggesting the PI3K/AKT signaling cascade is one of the critical players in melanomagenesis.

Mutations in cyclin-dependent kinase inhibitor 2A (CDKN2A) is common among human cancers including melanoma. It encodes two proteins, p16INK4a and p14ARF. P16 binds to cyclin-dependent kinases 4 and 6 (CDK4 and CDK6) and inhibits phosphorylation of Rb, which remains associated with the transcription factor, E2F. This inhibition of E2F prevents the G1/S transition in the cell cycle due to lack of the transcription of E2F targeted genes necessary for cell cycle progression. ARF complexes with MDM2, an E3 ubiquitin ligase that regulates the stability of p53 and therefore suppressing tumor growth, it also has been shown to be involved in the immune response, by modulating the tumor environment [25]. Therefore, mutation(s) in ARF dysregulate p53 function and play a role in tumor immune evasion.

CDK4 gene amplification, which is hypothesized to act as an independent oncogene, is more common in acral and mucosal melanoma than the CSID and NCSID cutaneous melanomas [9,10]. Mutations or deletions of PTEN are concurrent with mutations in BRAF but not in N-RAS [10]. Curtin and colleagues postulate that since N-RAS activates both the PI3K and MAPK pathway, while BRAF only activates the latter, it may suggest that in melanoma pathogenesis, somatic mutations activating one pathway require another event to activate other pathways [10].

In addition to various mutations in key component of signaling cascades, different types of RNAs have been shown to be involved in melanoma pathogenesis such as: miRNAs and lncRNAs [26,27]. Various miRNAs have been implicated in the processes of carcinogenesis leading to melanoma. miRNAs that have been shown to be upregulated in melanoma are: miR-101, -182, -221, -222, -106-363, -106a, -92, -196, -21, -156, -214, -30b, -30d and -532-5p. Those shown to be downregulated or lost in melanoma are: Let7a and b, miR-31, -125b, -148a, -211, -193b, -196a-1, -196a-2, and -203 [28].

Human melanoma cells were shown to have higher levels of the long non-coding RNA (lncRNA), SPRINGTLY, compared to normal human melanocytes [26]. Zhao and co-workers showed that stable clones isolated from introduction of SPRINGTLY in human melanocytes led to increased cell proliferation, colony formation, invasion, reduction in apoptosis, and development of a multinucleated dendritic-like phenotype. When siRNA was used to knockdown SPRINGTLY, a decrease in cell proliferation, invasion and increase in pro-apoptotic signals was detected [26].

## 4. Melanoma Detection Methods

Normally, melanoma is detectable by the irregular shapes in pigmented lesions on the skin; however, there are exceptions that are difficult to differentiate. Several techniques have been developed over the years to better detect melanoma before it advances to metastasis, which is crucial in improving the diagnosis, prognosis and treatment of melanoma patients [29].

Molecular assays such as: Fluorescent In-Situ Hybridization (FISH), Comparative Genome Hybridization (CGH), Quantitative Reverse Transcription Polymerase Chain Reaction (qRT-PCR), Next Generation Sequencing (NGS) and detection of exosomes have been shown to be of great value for melanoma detection. Melanoma tumor cells are characterized by certain chromosomal aberrations [29]. The FISH technology uses various fluorescent probes to distinguish between benign and melanoma tumors in unambiguous samples (87% and 95%, respectively), however its efficacy in ambiguous samples is yet to be demonstrated [29].

Another method of melanoma diagnosis is CGH, this method involves extracting normal and tumor DNA and fluorescently labeling each DNA sample with fluorophores of different colors [29]. The next step is to use the differentially labeled DNA probes and hybridize control DNA using either the metaphase chromosomes or DNA microarrays. The hybridization of the different colored fluorescence probes to the control DNA will allow the determination of any chromosomal region gain or loss based on the colors [29]. CGH allows for the better view of the genome allowing it to detect multiple chromosomal anomalies compared to FISH, which can only detect limited loci [29].

qRT-PCR and NGS are technologies which may have diagnostic values, however they are still in relatively early stages of development. qRT-PCR in a clinical setting will be used to determine the gene expression patterns of tumor samples. The tumor sample is given a score based on the gene expression measurement, and it will be categorized as a benign lesion or malignant melanoma based on the given score [29]. Furthermore, it could also allow physicians to distinguish between melanoma subtypes [29]. Additionally, the qRT-PCR is being utilized to better diagnose and design treatments for patients by way of predicting the metastatic risks in Stage I or II melanomas [29]. NGS could be a great diagnostic tool, due to its ability to sequence tumor DNA, not only in the coding regions that make up proteins, but also the regulatory regions that control the timing and levels of a given protein. These techniques will allow the clinicians to determine mutation-specific treatments; it will be interesting to see how these approaches impact the treatment outcome of human cancers [29].

## 5. Melanoma Treatments

### 5.1. Chemotherapy

The treatment for primary melanoma patients is surgical removal of the tumor(s). Treatment options for late stage melanoma patients include targeted drug therapies with or without radiation or immunotherapies. Many of the targeted therapies involve components of the MAPK/ERK pathway. A well-known target is the mutated BRAF protein and small molecule inhibitors have been developed against it [19]. The well-known BRAF inhibitors, vemurafenib/zelboraf (PLX4720/ PLX4032), have been shown to improve survival rates for many melanoma patients [19,30]. There has been great success with vemurafenib in clinical trials, which showed a response (measured as more than 90% reduction in active ERK) in greater than 50% of BRAF^V600E or V600K^ melanoma patients and the median overall survival rate was 16 months [30], however many of these patients can develop resistance to the inhibitor [3,19], likely due to the reactivation of the MAPK pathway or other mutations [3,19,30].

There have been various inhibitors developed against other components of the MAPK pathway. In vivo, the MEK inhibitor, selumetinib, has shown to reduce melanoma xenograft tumor growth [30,31]. Inhibitors of ERK have been shown to successfully inhibit the MAPK pathway in MEK-inhibitor resistant cells, since ERK is downstream of MEK [32]. It has not been possible to develop an inhibitor towards RAS [33]. Difficulties in inhibition of RAS has led to the development of other targets to indirectly inhibit the effector molecules of RAS by way of development of inhibitors of the RAF-ERK-MEK pathway such as farnesyltransferase, Rce1, lcmt1, PI3K-AKT-mTOR pathway and RalGEF-Ral pathway [33].

Malignant melanoma cells were shown to have higher levels of NOTCH signaling when compared to normal melanocytes, indicating its role in melanoma pathogenesis [34]. Under normal conditions, NOTCH signaling is required in the maintenance of melanoblast and melanocyte stem cells, however, in mature melanocytes, low or undetectable levels of NOTCH expression are found [34]. The gamma secretase inhibitor, GSI, was developed to target NOTCH signaling pathway and successfully suppresses NOTCH activation. However in phase 2 trials, only a modest responsiveness to GSI was observed in metastatic melanoma patients [34].

### 5.2. Combinatorial Chemotherapy

When melanoma patients undergo chemotherapy, the tumors are frequently known to adapt and become resistant against monotherapies. In order to increase the efficacy of therapeutic treatments, combination therapies are commonly administered. In preclinical studies, BRAF inhibitor-resistant mutated BRAF melanoma cells were developed as an experimental model system to mimic BRAF inhibitor-resistant melanoma patients and to study the mechanisms of resistance. These cells were treated with a combination of GSI and BRAF inhibitor, a reduction in cell growth and an increase in senescence were seen in these resistant cells, however, when they were weaned off GSI during the study, there was an increase in cell growth [34]. These pre-clinical findings suggest that in a combination therapy, which inhibits both MAPK and NOTCH pathways, may improve the efficacy of melanoma patients who develop BRAF inhibitor-resistance but both inhibitors must be present to sustain the anti-tumor progression responses.

### 5.3. Immunotherapy

Stage III melanoma patients along with surgery can be offered immunotherapy with interferon, which functions to enhance immune response, or the anti-cytotoxic T-lymphocyte-associated protein. For stage IV melanoma patients, they can undergo immunotherapy sessions in conjugation with other targeted drug chemotherapy, and in some cases radiotherapy [3]. Immunotherapies utilize the host’s immune system to elicit a tumor-specific immune response to combat cancer malignancies. Recent focus for immunotherapies has been on dendritic cell (DC)-based cancer vaccinations. DCs are of great interest in cancer because of their ability to uptake, process, and present antigens, which enables them to elicit an immune response. In the case of potential cancer vaccines, this immune response is against the cancer cells. Specifically, the use of dendritic derived-exosomes (DEXO), which are nanovesicles released from DCs, and have shown promise, as they contain the machinery required to activate potent antigen-specific immune response [35]. Damo et al. showed that DEXOs from DCs that were incubated with both a ligand for TLR-3 to stimulate the cytotoxic natural killer cells as well as the CD8+ cells, and melanoma antigens from necrotic B16F10 cells, the DEXOs were then injected into mice bearing B16F10 tumors, and resulted in a significant reduction in growth of the tumors [35].

Another focus of immunotherapy is targeting immune checkpoints that function as regulators of T-cell activation through receptor/ligand complexes [36]. Clinical trials have demonstrated that combining blocking cytotoxic T lymphocyte antigen-4 (CTLA-4) in conjunction with treatment of human monoclonal ipilimumab led to an increase in survival rates, a reduction of 34% death in a subset of advanced stage melanoma patients [36]. Another checkpoint target is the receptor/ligand, program death-1 (PD-1) and program death ligand-1 (PD-L1). Targeting this checkpoint pair was shown to have anti-tumor activity in melanoma patients [36]. PD-1 receptors interaction with its ligands, PD-L1 and PD-L2 in peripheral tissues, which induces a reduction in T-cell effector function and enhances apoptosis [36,37,38]. In metastatic melanoma, PD-L1 is upregulated along with tumor invading lymphocytes and IFN-γ production; suggest a process by which melanoma tumors evade immune system attack [36,37]. A phase I trial with the monoclonal anti-PD-1 antibody, nivolaumab, showed that 28% of the patients with advanced melanoma had a partial or complete response to treatment and out of those, 72% who received nivolaumab for more than a year were responsive to treatment that lasted for a year or more [36,39].

An alternative approach in conjunction with immunotherapies is natural compounds that have been explored as additional treatment options. Curcumin, a plant based chemical, has been shown to have anti-cancer effects including anti-angiogenic, pro-apoptotic and the ability to modify the immune system [40]. Curcumin, being a natural product, is generally less toxic than other synthetic drugs [40], but the bioavailability in the body for curcumin is low. Several groups are developing novel delivery systems such as nanoparticles, liposomes, micelles and phospholipid complex to increase its bioavailability [40]. Curcumin has been shown to mediate its anti-cancer effects by modulating the MST1, JNK, BIM-1, FOXO3, BCL-2, JAK-2/STAT-2, and BAX pathways, in in vitro models [40]. Specifically, in melanoma cells, curcumin was shown to induce apoptosis in a dose and time dependent manner [41]. In an advanced melanoma murine model, it was shown that treatment with amphiphilic curcumin–based micelle led to remodeled tumor microenvironment and enhance vaccine efficacy. A combination therapy using amphiphilic curcumin with vaccine therapy resulted in a downregulation of immunosuppressive factors as well as increased the efficacy of the vaccine treatment where there was a 7-fold increase in INF-y and increase in cytotoxic T-cell response [42].

## 6. Exosomes

Exosomes are naturally occurring small membrane enclosed microvesicles with a characteristic size of 30–120 nm in diameter, generated constitutively and released by various cell types [43]. They are shed from the surface of most cell types, and take with them membrane components and contents from the cytoplasm of the cells they are released from. Under normal cellular conditions, the release of exosomes accompanies normal cell growth and activation of some cellular functions, such as stimulation of T cell growth in vitro and induction of anti-tumor immune responses depending on specific cell types [44,45,46]. These vesicles contain various different DNA [47], miRNA [48,49], mRNA [50,51], and protein [52] and have the ability to enter circulation and act as messengers between cells [43]. The process involved in the formation of MVBs and exosomes was first reported in 1983 by Harding et al. [53], and confirmed in 1985 by Pan et al. [54] Using immunoelectron microscopy, they visualized the transfer of a transferrin receptor in reticulocytes from the cell surface to an early endosome, to a multivesicular endosomes, localized at the surface of the internal vesicles, then finally, fusion of these multivesicular compartments with the plasma membrane and the smaller vesicles bearing transferrin receptors were released into the extracellular environment [53,54]. More current studies have detailed exosome biogenesis in detail. Briefly, formation of exosomes is initiated by the inward budding of the cellular membrane to form an interluminal vesicle (ILV). The membrane of the ILV then buds inward undergoes scission to form the exosomes, which accumulate within the ILV, transforming it into a multivesicular body (MVB). The Endosomal Sorting Complex Required for Transport (ESCRT) machinery and associated proteins are largely responsible for this exosome biogenesis. In some cases ESCRT-independent mechanisms that exist to form MVBs, which rely on lipids, tetraspanins or heat shock proteins. The secretion of the exosomes involve RAB proteins that dock the MVB to the cellular membrane, which then results in the fusion of the two membranes, causing the release of the exosomes into the extracellular space. This membrane fusion is facilitates by the soluble NSF-attachment protein receptor (SNARE) complexes. This process is reviewed in detail by Kowal et al. [55]. These newly released exosomes have the ability to travel long distances within the body, or interact with local cells. The uptake of exosomes by target cells can occur in different ways; engulfment of the exosomes by the recipient cells often by clathrin-dependent endocytosis or, under certain conditions, lipid-dependent membrane fusion [56].

Exosomes contain a unique composition of proteins and nucleic acids that can vary depending on the cell type they are formed from, and the content generally reflects the function of that cell. Studies of exosomes from immature dendritic cells (DCs) [57,58], B lymphocytes [59,60], intestinal epithelial cells [61] and other cell types show that there are common, as well as cell-type specific proteins contained within the exosomes. Cell-type specific proteins within exosomes include Major Histocompatibility Complex (MHC) class I, which is usually found in exosomes derived from cells that are HLA-class I+. MHC class II proteins can be found on exosomes released from immune cells such as B lymphocytes, DCs and mast cells. These MHC II molecules present on immune-derived exosomes play direct roles in antigen-presenting and immune response induction [62]. While von Willebrand factor [63], perforin and granzymes [64] are found in platelet and cytotoxic T cell exosomes, respectively. The proteins that are found to be consistent across exosome types include chaperones (Hsc73 and Hsc90), subunits of trimeric G proteins, Tsg101, cytoskeletal proteins and tetraspanins such as CD9, CD63, CD81 and CD82 [57,58,61].

## 7. Exosomes and Cancer

There are many reviews that have covered the characterization and the formation of exosomes and their role in various diseases, including pathological infection [65], neurodegenerative diseases [66], liver disease [67], heart failure [68] and cancer [69]. Exosomes are more frequently released by tumor cells and may facilitate communication within the local microenvironment and the primary tumor [50,70,71,72], supporting tumor cell dissemination and early events in metastasis [73,74]. Here we briefly review exosomes from various cancer types, but focus mainly on melanoma-derived exosomes, and the studies that have focused on the role that those tumor derived exosomes play in the progression of the disease.

Exosomes may have the ability to influence cells that participate in metastasis by horizontal transfer of proteins, miRNAs and other molecules [75,76,77]. For example, exosomes derived from gastrointestinal stromal tumor (GIST) cells are enriched with oncogenic protein tyrosine kinase (KIT), are taken up by normal human myometrial smooth muscle cells (SMCs). This uptake of KIT-enriched exosomes causes intercellular KIT pathway activation, which results in a more adhesive phenotype in these recipient cells, and the secretion of matrix metalloproteinase 1 (MMP1). The secretion of MMP1 by SMCs causes an increase in the invasiveness of GIST cells [78]. Additionally, exosomes from hypoxic glioblastoma multiforme (GBM) cells contain hypoxia-regulated mRNAs and proteins, which are taken up by normoxic GBM cells and increase autocrine promigratory activation in the recipient cells. Those exosomes also interact with surrounding endothelial cells and pericytes causing paracrine induction of angiogenesis and resulting in tumor growth [79]. Melo et al. demonstrated that exosomes from cancer cell lines contain not only siRNAs, but also pre-siRNAs and the machinery required for the processing of those pre-siRNAs into siRNAs, which are then introduced into the target cells and silence endogenous mRNAs and promote tumorigenesis [80].

Exosomes from the cells within the surrounding environment also are involved in tumor progression by transferring macromolecules to target cells. For example, exosomes derived from fibroblasts were shown to influence the invasiveness abilities of breast cancer cells. Fibroblast-derived exosomes deliver Wnt11 protein, and cause the mobilization of the Wnt-planar cell polarity autocrine signaling, which results in increased invasive and metastatic characteristics of breast cancer cells [81]. Furthermore, glial exosomes have been shown to transfer siRNA, and induce specifically PTEN loss in breast cancer brain metastases resulting in enhanced tumor growth [49].

Recently, Peinedo et al. described the involvement of melanoma exosomes in tumor progression and the preparation of the pre-metastatic niche of future secondary tumor sites [82]. The pre-metastatic niche is created even before the tumor cell arrives at the site; cells from the immune system and the resident stromal cells all participate in the formation of a supportive environment for secondary tumor growth. Earlier studies demonstrated the ability of melanoma exosomes to “educate” bone marrow progenitor cells to be receptive of and support tumor cell growth and metastasis [82]. Once in circulation, exosomes have the capability to home towards the most common sites of metastasis and accumulate, this accumulation results in the leakiness of the vasculature, as well as recruitment of immune cells, two events involved in pre-metastatic niche formation [82].

Exosomes were shown to directly modulate the function of the immune system, which is critical in the progression of various different cancers. Breast cancer cell exosomes contain prostaglandin E2 (PGE2) and transforming growth factor beta (TGFβ), which induces myeloid cells in the bone marrow to become myeloid-derived suppressor cells (MDSCs) that promote tumor progression. MDSCs accumulate in the secondary lymphoid organs, blood and tumor tissues to provide supporting stroma and immune evasion [83]. Exosomes from both squamous cell carcinoma of the head and neck and dendritic cells activated human T-lymphocytes (Tregs that are more susceptible) and induce immunosuppression by interacting with the cell surface rather than internalization [84].

Exosomes have also been shown to promote cancer progression by multiple mechanisms of treatment resistance. Stromal cell exosomes (especially fibroblasts) increase the expression of pattern recognition receptors such as RIG-1 in breast cancer cells, which cause the upregulation of genes involved in treatment resistance [85]. Exosomal miRNAs from neuroblastoma cells mediate cellular crosstalk with co-cultured monocytes, downregulating target genes, and increasing telomerase activity, which is involved in chemoresistance [86].

Although these vesicles play important roles in the progression of cancer, numerous investigators suggested to use them as a promising non-invasive biomarker for cancer. Melo and colleagues identified particular proteins exclusively present on exosomes derived from malignant cells. They found that Glypican-1 (GPC1) is overexpressed in breast and pancreatic cancers and is found solely on exosomes derived from those malignant cells. Additionally, the level of GPC1+ exosomes in circulation correlates well with outcome after resection of pancreatic lesions [87]. Application of GPC1 as a prognostic marker to other cancers is unknown.

## 8. Exosomes and Melanoma

There are numerous ways that have been proposed for melanoma-derived exosomes to participate in the survival, proliferation and metastasis of melanoma.

### 8.1. Unique Composition of Melanoma Exosomes

The composition of exosomes often reflect the contents of the membrane and cytoplasm of the cells they are released from, which naturally would suggest that the contents of melanoma-derived exosomes are unique when compared to other tumor types, and even the normal cell counterpart. A study exploring this utilized 2D-PAGE analysis of both melanoma-derived exosomes and cell lysates from which they originated, strikingly different proteomic profiles were observed. The exosomes contained drastically less or were absent of lysosomal and mitochondrial proteins that were present in the cell lysates. In contrast, the proteins that were enriched in the exosomes of SK-MEL-28 and MeWo melanoma cell lines included p120 catenin, radixin, and immunoglobulin superfamily member 8 (PGRL) [88]. Other groups identified other melanoma-specific exosome proteins, including Rappa et al. who identified the presence of prominin-1/CD133 on exosomes released from these cells [89]. Prominin-1 is a transmembrane protein marker of both neural stem cells and hematopoietic progenitor cells, and is present in both melanoma cell lines and melanoma patient samples [90]. Exosomes that were prominin-1 positive were isolated and analyzed, and were found to contain, along with exosome-specific proteins, multiple pro-metastatic proteins, including CD44, MAPK4K, GTP-binding proteins, ADAM10 and Annexin A2 [90].

In clinical samples, exosomes were isolated from melanoma patients and found to contain higher concentrations of Melanoma Inhibitory Activity (MIA), a small protein secreted by malignant melanoma cells and S100B, a calcium binding protein involved in cell cycle progression and differentiation, expressed by melanoma cells, when compared to healthy volunteers [91]. Exosomes from liver perfusates of patients with metastatic uveal melanoma contained the protein melan-A [92].

Not only are certain protein enriched in melanoma exosomes, studies show a multitude of miRNAs and their expression profiles are specific to these vesicles. A study by Ragusa et al. revealed that exosomes derived from uveal melanoma patients contained a common miRNA in the exosomes, miR-146a, within the vitreous humor, as well as those circulating through the body [93]. Another miRNA in exosomes, miR-126b, was down-regulated only in patients with advanced melanoma compared to healthy donors [94]. In another study, Felicetti et al. demonstrated that the metastatic ability of melanoma cell lines was proportional to the amount of miR-222 within the exosomes.

In a clinical setting, exosomes derived from the plasma of sporatic metastatic melanoma patients displayed elevated levels of miR-17, -19a, -21, -126 and -149 compared to those with familial melanoma or healthy controls [95]. Additionally, there was no differential expression of miRNAs seen in familial melanoma patients and unaffected group [95]. These results suggest that in familial melanoma, genetic predisposition instead of miRNAs plays a critical component in the onset and progression of the disease, miRNAs could be used as a prognostic and diagnostic tools in patients with non-familial metastatic melanoma [95]. 

Not only do exosomes contain special proteins and miRNA, the lipid bylayer that encases the exosomal macromolecules are unique to melanoma exosomes. Melanoma exosomes are composed of lipid bilayers that contain a high concentration of sphingomyelin and high levels of tetraspanin proteins, which is hypothesized to be the determinant in the release of the exosomes [90].

Many studies have shown that exosomes from melanoma cells and of plasma from melanoma patients contain certain proteins and miRNAs. This has tremendous potential in diagnosis and prognosis of melanoma. For example, patients with an increased concentration of MIA in their circulating exosomes correlated with a shorter median survival rate [91]. Additionally, detection of metastasis has great potential using these vesicles; a group found that the profiling of exosomal miRNA from metastasis in the liver showed differences when compared to the primary tumor [92]. Understanding the unique composition of melanoma exosomes has the potential in detecting lesions and influencing the course of treatment of melanoma patients, and continues to contribute to our understanding of the progression of the disease.

### 8.2. Promotion of Aggressive Behavior in Cancer Cells by Exosomes

The unique composition of exosomes released from melanoma cells may contribute to their ability to manipulate other cells. Melanoma cell derived exosomes have been shown to induce tumorigenesis or induce pro-metastatic behaviors in other melanoma cells, as well as normal cells. These exosomes seem to contain different determinants from the cells they originate from, which may be involved in promoting metastatic behavior. For example, miR-222 has been shown to play a tumorigenic role in melanoma, by its ability to induce the PI3K/AKT pathway and this miRNA can be transferred from the exosomes to the recipient cell and cause subsequent induction of the PI3K/AKT pathway [96]. Interestingly, Nieto and colleagues identified several unique proteins only found in exosomes from highly metastatic melanoma cell lines. These proteins are known to be involved in cell motility, angiogenesis and immune responses, suggesting the transfer of pro-migratory proteins from the highly metastatic exosomes to the less aggressive ones [97].

Indeed, the characteristics of a cell line that exhibits a metastatic phenotype has the ability to transfer proteins to recipient cells that gain some of those characteristics. For example, exposure of mesenchymal stem cells (MSCs) to exosomes from Prominin-1-positive melanoma cells resulted in an increased invasiveness of the MSCs [90]. Xiao et al. also found that normal melanocytes could gain the ability to invade when incubated with exosomes from melanoma cells [98]. The pro-metastatic protein, Met72, was detected in the highly metastatic clone of B16 melanoma cells (B16-10). B16-10 exosomes express Met72, and can be taken up by the poorly metastatic clone of B16, B16-F1, which then begins to express Met72 and exhibit metastatic activity similar to B16-10 cells [99]. Another example of a pro-metastatic phenotype transferred to another cell via exosomes involves WNT5A. In malignant melanoma cells WNT5A induces a calcium-dependent release of exosomes that contain immuno-modulatory and pro-angiogenic factors involved in metastasis that have the ability to induce immune suppression and angiogenesis [100]. It is hypothesized that melanoma exosomes induce the release of vascular endothelial cell derived tumor necrosis factor alpha (TNF-α), which causes the lymphatic endothelial cells to tolerate tumor growth within the nodes [101].

Interestingly, exosomes from other cell types have the ability, through exosome release, to increase pro-metastatic phenotypes. Exosomes released by adipocytes contain proteins involved in fatty acid oxidation (FAO), which are only found in these exosomes. They can be taken up by melanoma cells induced elevated FAO levels and increased in migration and invasion [102]. In another report, neural cell exosomes were shown to have the ability to affect the morphology and physiology of melanoma cells including activation of MAPK pathway within the cell, modulating melanogenesis and dendrite-like outgrowths of the cells, supporting the notion that exosomes from one cell type is able to influence the differentiation and cell signaling of another [103].

### 8.3. Melanoma Exosomes Manipulate Primary Tumor Microenvironment

Melanoma cell-derived exosomes have been shown to manipulate primary tumor microenvironment by: (1) supporting the epithelial-to-mesenchymal transition (EMT) of the cells in the melanocytic microenvironment, promoting metastasis, through autocrine/paracrine signaling activating the MAPK pathway. miRNAs involved in this transition, let-7i, mir191 and let-7a, were shown to be present in circulating exosomes from stage 1 melanoma patients but not in exosomes from non-melanoma patients [104]; (2) Affecting the differentiation of immune cells by enhancing the maturation of dendritic cells and T-cell proliferation [105]; (3) Activating macrophages when treated with melanoma-derived exosomes and exhibit a different cytokine and chemokine profile than when exposed by other activators such as LPS or IL-4 [105]; and (4) increasing migration of endothelial cells and inducing angiogenesis, perhaps by transfer of miR-9 from the melanoma cells to endothelial cells via exosomes [106].

### 8.4. Melanoma Exosomes Manipulate the Pre-Metastaic Niche

In addition to the ability of melanoma-derived exosomes to affect the local cellular environment, these exosomes also have been shown to travel throughout the body and accumulate in distant organs. Peinado et al. demonstrated the distribution and metastatic potential of B16-derived melanoma exosomes in the lung [82], and was supported by results from Morishita and colleagues [107], where they demonstrated the distribution of inoculated radio-labelled B16BL6 murine melanoma-derived exosomes throughout the body, and found that after a very short half-life in circulation, these radio-labelled exosomes accumulated in the lung, spleen and liver. Similar observations were made earlier with the gLuc-LA-coupled B16BL6-exosomes [108].

The premetastatic niche (PMN) is the site of possible secondary metastasis. This microenvironment is made up of multiple different cell types, including fibroblasts, infiltrating immune cells, endothelial cells, and other cells that comprise the blood and lymph vessels. These and the extracellular matrix must create a supportive microenvironment for the arrival, growth and establishment of a secondary tumor from the circulating tumor cell destined to arrive there [109].

Melanoma exosomes may play an important role in the formation of the PMN. They have been shown to induce vascular leakiness at pre-metastatic sites, an event that is important in the formation of the niche. Exosomes injected into xenograft tumor bearing mice showed changes in mRNA profiling of the lungs, mainly in those that are involved in various steps in pre-metastatic niche formation. Bone marrow progenitor cells also accumulated in pre-metastatic niches [82]. These exosomes probably induce molecular signals that help melanoma cells prepare sentinal lymph nodes for metastasis, recruit other critical molecules, ECM deposition and vascular proliferation within the nodes [74].

Tumor-derived exosomes are hypothesized to also be involved in manipulating interactions between the origin tumor cells, and the surrounding tissue stroma to promote malignancy. Specifically, those exosomes have the ability to interact with immune cells, which then help manipulate the microenvironment to be conducive for metastatic growth. For example, human melanoma and colorectal carcinoma-derived microvesicles have been shown to promote the differentiation of monocytes to myeloid-derived suppressor cells that support the growth of the tumor and the ability to escape immune surveillance [110]. Other immune cells have been shown to interact with melanoma exosomes; RNA from either melanoma cells or Lewis lung carcinoma cell-derived exosomes are taken up by lung epithelial cells and result in activation of Toll-like receptor-3 (TLR3) in these cells and causes the infiltration of neutrophils. This infiltration promotes pre-metastatic niche (PMN) formation in the lung. TLR3-deficient mice do not form lung metastases and have a reduction in PMN formation due to a decrease in neutrophil infiltration [111].

Melanoma exosomes have also been implicated in the promotion of angiogenesis by regulating endothelial cells from a distance, and manipulate cytokine expression profiles to establish an immunosuppressive environment [73].

### 8.5. Resistance to Treatment

Commonly, when patients are undergoing various treatment regiments, the tumor initially shrinks, then seems to spontaneously develop resistance and begins to resume growth, regardless of continuation of treatment. There have been recent studies suggesting the involvement of melanoma-derived exosomes in treatment resistance. Melanoma cells also have the ability to create an acidic microenvironment. This reduction in pH is a mechanism of inducing resistance for these cells from cisplatin treatment. When the cells are co-treated with proton pump inhibitors and cisplatin, exosome release is reduced, in addition to a higher pH, and an increased amount of uptake of cytotoxic cisplatin by the cells [112]. Melanoma cells have also been shown to accumulate chemotherapeutic agents within vesicular compartments and release them in exosomes, as shown by Chen et al. with cisplatin treatment [113]. Melanosome release is enhanced in the presence of cisplatin where they are exploited for cisplatin removal from the cell [113].

## 9. Conclusions

**Figure 1:**
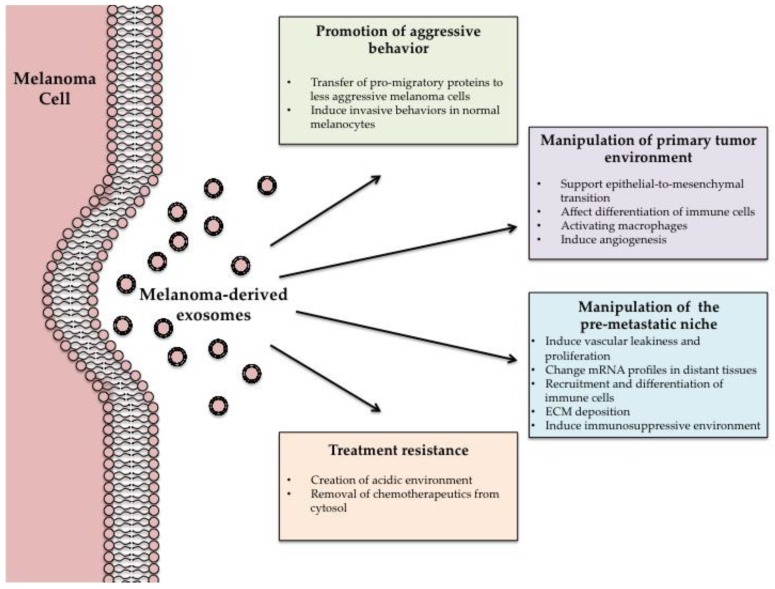
Roles of Exosomes in Tumor Development and Progression: a summary of the proposed roles/functions mediated by melanoma exosomes in tumor development and progression.

Migration, invasion and pre-metastatic niche formation are all important events that occur in metastasis of a primary tumor, and understanding all aspects of this process is essential to prevent cancer-related deaths, including melanoma. This review explored the significance, causes and treatments of melanoma, and summarized recent publications that highlight the role of melanoma-derived exosomes in the progression of the disease (summarized in Figure 1). Not only do the tumor-derived exosomes participate as cellular messengers, these vesicles have been shown to be involved in various steps that are essential for a successful melanoma metastasis. A few different melanoma exosome-specific proteins found in the circulating exosomes of patients have been shown to correlate with prognosis; this evidence shows the great possibilities of the use of exosomes in cancer detection and estimating prognosis. The field of exosome research in cancer progression is expanding, and being explored as a target for therapy as well as a tool to deliver anti-cancer drugs to tumors.
